# Intraoperative Hemodynamic Management and Cerebral Protection During Awake Craniotomy: Evidence‐Based Approaches

**DOI:** 10.1155/anrp/5356459

**Published:** 2026-08-29

**Authors:** Marc Knezevic Maragh, Alanna Pagan Garcia, Yuliia Oliinyk, Jayottri Acharjee, Alex Emmanuel Binoy, Feaven Tewfik, Khalil Garada, Loveleen Johal, Nafrin Kormath, Abdulaziz Alkhalidi, Nusaiba Ali, Khalé Reyes

**Affiliations:** ^1^ Department of Medicine, Ross University School of Medicine, Bridgetown, Barbados, duhs.edu.pk; ^2^ Department of Medicine, Universidad Autónoma de Guadalajara, Guadalajara, Mexico, uag.mx; ^3^ Department of Medicine, Odesa National Medical University, Odesa, Ukraine, duhs.edu.pk; ^4^ Department of Medicine, Polytechnic University of Marche, Ancona, Italy, univpm.it; ^5^ Department of Medicine, Hywel Dda University Health Board, Aberystwyth, UK, duhs.edu.pk; ^6^ Department of Medicine, St. George’s University School of Medicine, St. George’s, Grenada, sgu.edu; ^7^ Department of Neurosurgery, University of Debrecen, Debrecen, Hungary, unideb.hu; ^8^ Department of Medicine, MES Medical College, Perinthalmanna, India, duhs.edu.pk; ^9^ Department of Medicine, University College Dublin School of Medicine, Dublin, Ireland, duhs.edu.pk; ^10^ Department of Medicine, University of Leicester, Leicester, UK, le.ac.uk; ^11^ Department of Medicine, Ross University Medical School, Bridgetown, Barbados, duhs.edu.pk

**Keywords:** awake craniotomy, cerebral protection, hemodynamic, intraoperative

## Abstract

Awake craniotomy is a standard neurosurgical technique that enables real‐time assessment of brain functions during tumor resection, with high success rates. Although highly effective, the procedure is associated with hemodynamic disturbances, including blood pressure variability and impaired cerebral perfusion, compromising intraoperative safety. A focused literature search of PubMed, Embase, and Google Scholar was performed to identify relevant studies on intraoperative hemodynamic management during awake craniotomy. Findings were synthesized narratively to summarize current evidence, highlight emerging strategies, and identify knowledge gaps. Hypertension (8%–34%) was the most common disturbance, often triggered by painful surgical stimuli or emergence from sedation, with repeat craniotomy, impaired autoregulation, and glioma pathology as additional risk factors. Hypotension (10%–11%) was linked to perioperative stroke and mortality. Cerebral hypoperfusion contributed to poor outcomes, with stroke up to 23% and neurological morbidity in 68% of patients. Preventive strategies, such as scalp blocks, individualized blood pressure targets, and the use of dexmedetomidine and midazolam, improved intraoperative stability, while short‐acting agents, including esmolol and labetalol, were preferred for rapid control. Advanced monitoring modalities, including arterial lines, near‐infrared spectroscopy, and cerebral autoregulation indices, enhance safety and support accurate functional mapping. Hemodynamic instability during awake craniotomy influences outcomes. Current evidence supports multimodal prevention, tailored blood pressure control, and short‐acting pharmacological agents; practice variation and the lack of randomized trials limit standardization. Looking ahead, advanced monitoring and emerging AI systems may provide new opportunities to enhance cerebral protection and optimize patient safety. This review summarizes evidence on intraoperative hemodynamic control in awake craniotomy, emphasizing strategies that enable maximal safe resection while preserving neurological function.

## 1. Introduction

Awake craniotomy is a neurosurgical technique that enables real‐time neurological evaluation during the surgical removal of lesions within or near functional brain areas, with the aim of preserving speech and motor function [1]. By maintaining patient responsiveness during surgery, this technique allows intraoperative functional assessment of eloquent cortical regions that would otherwise be inaccessible under general anesthesia [1, 2].

Several anesthetic strategies are currently used for awake craniotomy. These include procedures performed entirely under conscious sedation, asleep–awake techniques, and the asleep–awake–asleep (SAS) approach, which is frequently employed to improve patient comfort during the initial operative stages [2, 3]. Successful anesthetic management during functional mapping depends on achieving patient comfort without compromising cooperation, spontaneous respiration, or cardiovascular stability, regardless of the technique employed [2, 4].

Awake craniotomy does not appear to be associated with a procedure‐specific hemodynamic disorder. Instead, intraoperative blood pressure fluctuations reflect common perioperative physiologic responses related to anxiety, surgical stimulation, variations in anesthetic depth, and sympathetic activation [2, 5]. These physiologic changes are clinically relevant because cerebral perfusion is highly dependent on stable systemic hemodynamics during neurosurgical interventions [5].

Both hypotension and hypertension may adversely affect cerebral perfusion during awake craniotomy. Episodes of hypotension can reduce cerebral blood flow (CBF) and oxygen delivery, whereas excessive hypertension may increase intracranial pressure (ICP) or interfere with surgical exposure [2, 4]. As a result, careful intraoperative blood pressure control is considered a fundamental component of anesthetic management in these procedures [2].

Cerebral autoregulation maintains CBF over a range of arterial pressures, though its effectiveness varies among individuals and may be compromised by intracranial pathology or changes in carbon dioxide levels [6]. Under these conditions, strict reliance on rigid blood pressure thresholds may be inappropriate, and increasing emphasis is placed on tailoring hemodynamic goals to each patient’s baseline blood pressure and intraoperative physiologic conditions to limit the risks of cerebral ischemia or hyperperfusion [3, 7].

Recent advances in neuroanesthesia have contributed to improved hemodynamic stability during awake craniotomy. In particular, the use of *α*2‐adrenergic agonists, such as dexmedetomidine, has been associated with preserved respiratory drive, improved patient cooperation, and reduced sympathetic responses during surgery [4, 7, 8]. Despite these developments, optimal strategies for intraoperative hemodynamic management and cerebral protection during awake craniotomy remain under investigation.

This narrative review summarizes current evidence on intraoperative hemodynamic management during awake craniotomy, with a focus on blood pressure regulation, cerebral perfusion, sedative selection, and monitoring strategies that may influence neurological outcomes.

## 2. Literature Search Strategy

A narrative review was conducted to evaluate intraoperative hemodynamic management and cerebral protection strategies in awake craniotomy. A focused literature search of PubMed, Embase, and Google Scholar was performed on August 24, 2025, to identify English‐language studies published between January 2010 and August 2025 using relevant keywords and MeSH terms, including *craniotomy*, *awake craniotomy*, *hemodynamics*, and *CBF*. Priority was given to clinical trials, cohort studies, systematic reviews, meta‐analyses, and landmark observational studies that contributed substantially to current understanding of the topic. Studies were selected based on their clinical relevance and methodological quality, and the evidence was synthesized narratively to summarize current practice, emerging strategies, and knowledge gaps. Because definitions of intraoperative hypotension varied across studies (including MAP < 60 mmHg, MAP < 65 mmHg, MAP < 70 mmHg, SAP < 80 mmHg, and SBP < 90 mmHg), an operational threshold of MAP < 65 mmHg was used for discussion purposes. However, original study‐specific definitions were preserved when reporting individual findings, and no attempt was made to retrospectively reclassify outcomes under a single threshold.

## 3. Review

### 3.1. Hypertension

#### 3.1.1. Incidence, Triggers, and Physiological Relevance

Intraoperative hypertension is among the most frequently reported complications during awake craniotomy. Reported rates range from 8% to 34%, depending on anesthetic technique, monitoring approach, and patient population [9–12]. Several clinical series have identified hypertension as the leading intraoperative hemodynamic event, more frequent than desaturation or seizures [10, 11]. Risk factors include painful surgical stimuli, such as scalp pinning and dural opening, which can provoke sharp blood pressure increases when analgesia is inadequate [13, 14]. Hypertensive episodes have also been reported during emergence from sedation, with shorter emergence times associated with fewer adverse events [15]. Repeat craniotomies further increase vulnerability because adhesions and fragile vasculature increase the likelihood of bleeding when pressure rises [16].

**FIGURE 1:**
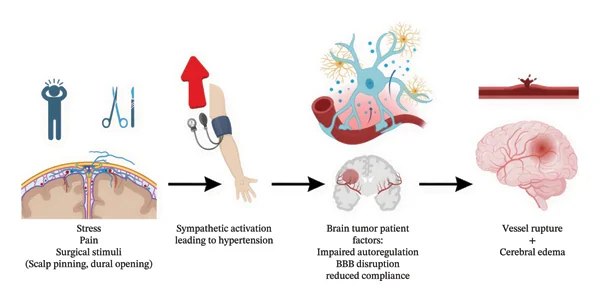
Pathophysiological cascade leading to intraoperative hypertension in awake cranioto*my*. Pain and surgical stress trigger sympathetic activation, elevating systemic blood pressure. In patients with gliomas, impaired autoregulation, disrupted blood–brain barrier, and reduced intracranial compliance amplify vulnerability, predisposing to hemorrhage and cerebral edema [14, 16, 17, 20].

The mechanism of intraoperative hypertension during awake craniotomy is primarily related to sympathetic activation triggered by pain, stress, and direct surgical stimulation [14, 17]. Intraoperative seizures during cortical stimulation may also provoke transient hypertension and hemodynamic instability through sympathetic activation and catecholamine release [14, 18, 19]. In patients with brain tumors, these responses are more problematic because cerebral autoregulation is often impaired, limiting the brain’s ability to compensate for sudden increases in perfusion pressure. Disruption of the blood–brain barrier (BBB) in malignant gliomas further increases vulnerability, as leaky vasculature allows fluid and proteins to extravasate, promoting cerebral edema [20]. Finally, the reduced intracranial compliance means that even modest elevations in pressure can cause disproportionate shifts in intracranial dynamics, placing fragile tumor vessels at higher risk of rupture and bleeding [16], as shown in Figure 1. These physiologic factors explain why close blood pressure control is consistently emphasized across clinical series of awake craniotomy.

Although pain and inadequate analgesia remain the primary drivers of sympathetic activation, several surgical factors can also contribute to hypertensive episodes during awake craniotomy. During the awake phase, direct cortical and subcortical stimulation used for functional mapping may produce transient physiological stress responses, particularly when repeated stimulation provokes intraoperative seizures or requires interruption of testing [21]. In addition, prolonged language or motor mapping and the need to remain in a fixed surgical position for several hours can increase patient discomfort and psychological stress despite adequate sedation [21]. These factors reinforce that blood pressure fluctuations during awake craniotomy are not solely determined by anesthetic management but also by what is happening during the surgical procedure, making continuous communication between the neurosurgical and anesthesia teams essential for anticipating periods of increased hemodynamic stress and intervening promptly when needed.

### 3.2. Clinical Implications

#### 3.2.1. Risk of Postoperative Hemorrhage

Uncontrolled intraoperative hypertension during awake craniotomy carries important consequences for both immediate and long‐term outcomes. In glioma patients, higher systolic pressures have been linked with an increased risk of early postoperative hemorrhage, supporting the practice of strict postoperative control [22].

#### 3.2.2. Neurological Morbidity and Functional Outcomes

In larger series, intraoperative instability, defined by events, such as hemodynamic fluctuations requiring vasoactive support, respiratory insufficiency, seizures, or agitation requiring intervention, was commonly observed under monitored anesthesia care (MAC) and, in some cases, contributed to conversion to general anesthesia, which can compromise surgical mapping and delay recovery [18]. Beyond single‐center reports, systematic reviews of glioma and insular glioma surgery have also shown that postoperative morbidity commonly includes hemorrhage, cerebral edema, and seizures [20]. These complications are among the main concerns when blood pressure is not well controlled, since hypertensive episodes can increase bleeding risk and worsen cerebral swelling.

#### 3.2.3. Impact on Intraoperative Mapping and Surgical Stability

Meta‐analyses of eloquent glioma resections have shown that intraoperative complications, of which hypertension is a leading contributor, were associated with higher postoperative morbidity, with complication rates of 15% in awake procedures compared with 22% in general anesthesia [23]. Together, the evidence indicates that intraoperative hypertension is a key determinant of both surgical success and postoperative function, making its management central to safe awake craniotomy practice.

Functional mapping itself can influence intraoperative hemodynamic variability and should be considered when discussing hemodynamic management. Awake craniotomy depends on continuous patient cooperation during language, motor, and cognitive testing, often requiring repeated cortical or subcortical stimulation while balancing sedation and analgesia [2, 4]. These periods of stimulation, patient anxiety, discomfort, or agitation can provoke sympathetic activation, resulting in transient hypertension, tachycardia, and blood pressure variability that may interfere with surgical stability and mapping conditions [2, 18]. Conversely, excessive hypotension or impaired cerebral perfusion may diminish patient responsiveness, compromise the accuracy of intraoperative neurological assessment, and increase the risk of ischemic injury in functionally critical brain regions [2, 4]. Therefore, stable hemodynamic control during functional mapping serves not only a neuroprotective role but is also essential for preserving mapping accuracy and maximizing safe tumor resection [18].

### 3.3. Evidence‐Based Management

#### 3.3.1. Preventative Strategies

**FIGURE 2:**
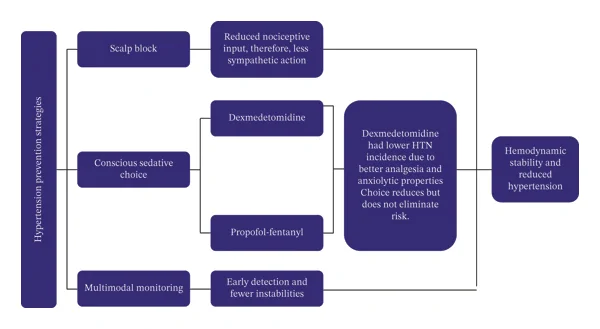
Prevention of intraoperative hypertension during awake craniotomy. Hypertension arises primarily from sympathetic activation triggered by pain, anxiety, and direct surgical stimulation [13]. Preventive strategies include scalp nerve blocks to blunt nociceptive input [24], careful sedative selection (dexmedetomidine providing more stable hemodynamics than propofol through its combined analgesic and anxiolytic effects) [24, 29], and multimodal monitoring to anticipate and manage hemodynamic responses. These measures together reduce hypertensive episodes and promote intraoperative stability [17, 25].

Prevention of intraoperative hypertension in awake craniotomy relies on minimizing painful stimuli and selecting anesthetic regimens that promote hemodynamic stability. Preanesthetic scalp nerve blocks reduce intraoperative pain and help maintain more stable pressures [24]. Their value is especially clear in the early surgical stages, where stimulation is otherwise likely to provoke a rise in blood pressure [13]. Clinical series also report more stable hemodynamics when dexmedetomidine is combined with scalp blocks [25]. The safety of repeated local anesthetic administration has been demonstrated, with prospective studies of ropivacaine showing that serum concentrations remain well below toxic levels even when multiple sites are injected [26]. These measures together reduce hypertensive episodes and maintain intraoperative stability, as shown in Figure 2.

#### 3.3.2. Sedative Selection

The choice of sedative agent is equally important. Dexmedetomidine is widely used because it maintains cooperation while generally preserving stable blood pressure [25]. However, loading doses of 1 μg/kg can provoke transient hypertension if administered rapidly and are best avoided or given slowly [27]. Propofol more commonly lowers systemic vascular resistance and can lead to hypotension [28]. Yet, retrospective data show that hypertension was reported more frequently with propofol compared with dexmedetomidine (35% vs 15%) [29]. This likely reflects that propofol does not control stress responses to stimulation as well as dexmedetomidine. Randomized trials further demonstrate that both propofol–dexmedetomidine and propofol–remifentanil regimens can still be associated with hypertension, showing that sedative choice reduces but does not eliminate the risk [30].

Remimazolam, an ultrashort‐acting benzodiazepine, has emerged as a potential alternative sedative for awake craniotomy, most commonly within SAS techniques [31]. Compared with propofol, peri‐induction data from general surgical populations suggest that remimazolam is associated with less hypotension and bradycardia while allowing rapid recovery, although some studies have reported a higher incidence of intraoperative hypertension, indicating that its hemodynamic profile may vary depending on dosing strategy and clinical context [32]. Direct evidence on remimazolam in awake craniotomy remains limited. Although one prospective randomized trial compared remimazolam–flumazenil with propofol during awake craniotomy and reported faster arousal with improved intraoperative task performance and no significant intraoperative complications [33], no head‐to‐head trial has directly compared remimazolam with dexmedetomidine in this specific setting. Therefore, any comparison between remimazolam and dexmedetomidine must currently be extrapolated from adjacent perioperative sedation studies, where remimazolam appears to offer comparable blood pressure stability, lower bradycardia risk, and faster sedation or recovery, but may carry context‐dependent risks, such as respiratory depression [34, 35]. Future randomized trials directly comparing remimazolam, dexmedetomidine, and propofol in awake craniotomy are needed to clarify their relative effects on hemodynamic stability, respiratory safety, arousal quality, patient cooperation, and mapping performance.

In the setting of awake craniotomy, the most clinically relevant advantage of remimazolam is its predictable and rapid awakening, which can be actively reversed with flumazenil to facilitate timely neurological assessment during cortical mapping [31]. Compared with dexmedetomidine, which provides cooperative sedation and sympathetic attenuation but is frequently associated with bradycardia and hypotension, remimazolam may allow a more controlled transition between sedation and wakefulness [36]. Additional comparative experience will be useful in determining whether these proposed advantages result in more consistent hemodynamic control, improved mapping conditions, or fewer conversions to general anesthesia.

#### 3.3.3. Pharmacologic Interventions

Management of intraoperative hypertension in awake craniotomy relies on clear thresholds for intervention and the use of short‐acting antihypertensive drugs. Most studies consider systolic pressures above 150–160 mmHg or more than 20% above baseline as requiring treatment [12, 24]. Postoperatively, maintaining systolic pressures below 140 mmHg has been associated with reduced hemorrhage risk in glioma patients [22]. When hypertension requires active treatment, short‐acting intravenous drugs are preferred because they allow blood pressure to be corrected quickly and then readjusted if needed. In a series of 74 awake craniotomies, the medications and dosing ranges used included esmolol 20–100 mg, labetalol 5–30 mg, and nitroglycerin 0.1–1.4 mg given in small boluses, with noninvasive pressures cycled every 3–5 min and a perioperative systolic goal below 150 mmHg [37]. While this shows nitrates have been used in practice, other institutions caution against their routine use because they may increase cerebral blood volume and ICP [38]. For this reason, short‐acting beta‐blockers, such as esmolol and labetalol, remain the preferred first‐line agents in most protocols in this setting [37, 38].

Other vasoactive agents employed in clinical practice include calcium channel blockers (nicardipine, nifedipine, and diltiazem), ACE inhibitors (lisinopril), and alpha‐1 antagonists (urapidil). Alpha‐2 agonists (clonidine and dexmedetomidine) offer the distinct advantage of dual therapeutic effects, providing both blood pressure reduction and adjunctive analgesia and sedation, making them particularly valuable in the awake craniotomy setting [38, 39]. Extrapolating from postcraniotomy emergence hypertension data, nicardipine demonstrates clear advantages over beta‐blocker monotherapy. A prospective randomized trial found nicardipine superior to esmolol in controlling hypertension, achieving more rapid blood pressure control with significantly fewer rescue interventions required (5% failure rate versus 55% with esmolol) [40].

Current practice is largely driven by clinician preference, with antihypertensive selection guided by specific clinical needs and the constraints of maintaining patient cooperation during intraoperative neurological monitoring [37, 38].

#### 3.3.4. Monitoring Recommendations

Blood pressure should be closely monitored, with noninvasive measurements repeated every 3–5 min, and invasive arterial monitoring considered for patients at higher risk or with anticipated instability [37]. Reports from larger series also emphasize that proactive monitoring and anticipation of hemodynamic events reduces the likelihood of instability and conversion to general anesthesia [18]. Management should therefore address both persistent hypertension and the sharp rises that occur during stimulating phases of the procedure, so that interventions can be given promptly and complications avoided.

### 3.4. Evidence on Hemodynamic Strategies

Overall, most available evidence supports the combined use of scalp nerve blocks and *α*_2_‐agonist sedation, particularly dexmedetomidine, as the most consistent strategies for maintaining hemodynamic stability during awake craniotomy. Scalp blocks attenuate nociceptive input at the source, while dexmedetomidine suppresses sympathetic activation without compromising respiratory drive, allowing steady pressures during stimulation and emergence. Despite these advantages, clear thresholds for blood pressure correction, optimal dosing, and preferred combinations with adjunct agents remain poorly defined. The heterogeneity among current studies, ranging from anesthetic protocols to MAP targets, limits the development of standardized algorithms. Addressing these gaps through larger controlled trials and standardized outcome measures would help establish more reliable guidance. Until stronger evidence emerges, individualized management guided by baseline hemodynamics, patient comorbidities, and real‐time physiological monitoring remains the most reasonable and clinically relevant approach.

## 4. Hypotension

### 4.1. Incidence and Physiological Relevance

Intraoperative hypotension is a major concern in neurosurgery, with incidence rates reaching up to 65% in emergency cases, whereas elective awake craniotomy generally demonstrates a lower frequency. While not specific to awake craniotomy, these findings highlight the vulnerability of neurosurgical patients and emphasize the importance of maintaining stable blood pressure for optimal cerebral perfusion and mapping accuracy [41]. Large database analyses also suggest that even brief intraoperative hypotension (mean arterial pressure [MAP] < 65 mmHg) is independently associated with increased perioperative stroke and mortality, further emphasizing the need for strict cerebral perfusion control in neurosurgical patients [42]. While scalp nerve blocks prevent hypertensive responses to surgical stimulation, they may also contribute to baseline hypotension by reducing sympathetic tone, requiring careful fluid management [43]**.**

#### 4.1.1. Hemodynamic Fluctuations in Awake and Asleep–Awake Craniotomy

Motor mapping during craniotomy can be performed in both awake and asleep patients, but complications, such as seizures, respiratory depression, and hemodynamic instability, are reported in both approaches. In a 238‐patient series, asleep–awake cases showed marked systolic arterial pressure (SAP) fluctuations, with hypertension (27%), seizures (16%), and hypotension (10%) among the most frequent events [44]. Across two studies, awake craniotomy was associated mainly with hemodynamic fluctuations and seizures. Using the original definitions from each study, hypotension (MAP < 60 mmHg) was observed in 11.1% of patients in the Mahajan et al. cohort, whereas the prospective series defined hypotension as SAP < 80 mmHg and reported a 10% incidence; hypertensive episodes were absent in the former (≈0%) but occurred in 27% of asleep–awake cases in the latter (SAP > 170 mmHg) [27, 44]. The novel enhanced recovery after neurosurgery (ERANS) protocol is a multimodal enhanced recovery pathway for awake craniotomy that combines preoperative counseling and preparation, intraoperative monitoring with tailored anesthetic management, and postoperative interventions to optimize safety, comfort, and recovery. While preliminary data suggest enhanced protocols may reduce hypotension rates, larger studies are needed to validate these findings [45].

#### 4.1.2. Surgical and Procedural Contributors to Hemodynamic Instability

Although intraoperative hypotension during awake craniotomy is frequently attributed to anesthetic depth or sedative selection, surgical and procedural factors may also contribute to hemodynamic instability, particularly during direct electrical stimulation (DES), where stimulation thresholds and afterdischarge thresholds vary across cortical regions and between institutions [46]. Stimulation burden is clinically relevant, as longer total stimulation duration, greater average stimulation duration, and a higher number of stimulations have been significantly associated with afterdischarge incidence, with total stimulation time > 145 s and > 60 stimulations linked to increased afterdischarge risk [47]. Mapping‐related intraoperative seizures are also multifactorial, with Penfield stimulation and diffuse pathology associated with higher seizure risk, and these events may interrupt mapping, reduce patient cooperation, and destabilize blood pressure or respiration [47]. Cold saline or Ringer lactate irrigation is mainly described as a treatment for stimulation‐induced seizures, serving as a protective measure to terminate seizure activity and facilitate the continuation of cortical mapping [48].

Subcortical mapping and prolonged language testing may further increase physiological stress, as awake testing typically lasts approximately 1.5–2 h before patients may become distressed or fatigued, potentially reducing cooperation and increasing sedative or vasoactive requirements [49]. Patient positioning represents another important procedural factor, since neurosurgical positions can influence ICP, cerebral hemodynamics, venous return, and cardiovascular stability [50]. Finally, deep tumor resection, vascular manipulation, and brain retraction may cause regional ischemia even when systemic blood pressure appears adequate, with proposed mechanisms including direct vascular injury, coagulation, vasospasm, and arterial kinking due to retraction [51]. Overall, these factors indicate that hypotension and cerebral hypoperfusion during awake craniotomy are not exclusively anesthetic concerns, but require coordinated surgical and anesthetic planning around stimulation strategy, mapping duration, patient positioning, and avoidance of vascular traction [52].

### 4.2. Clinical Implications

In awake craniotomy, hypotension carries significant clinical implications, as impaired cerebral perfusion can increase the risk of ischemia or infarction [53]. Continuous blood pressure monitoring remains essential, and invasive arterial lines are particularly recommended in patients with baseline hypertension, large tumor burden, or anticipated hemodynamic instability, as these groups are at the highest risk of requiring active blood pressure management [22] A recent meta‐analysis of awake craniotomy under MAC reported cardiovascular complications, including hypotension, hypertension, and arrhythmias in 21% of patients (95% CI: 10%–32%, *p* < 0.001; I^2^ = 88.0%), underscoring the high prevalence and clinical importance of hemodynamic instability in this setting [8]. Despite the recognized clinical significance of hypotensive episodes, the current literature has limited specific data on the direct outcomes and long‐term consequences attributable to intraoperative hypotension during awake craniotomy procedures.

### 4.3. Management

#### 4.3.1. Pharmacological Strategies

**FIGURE 3:**
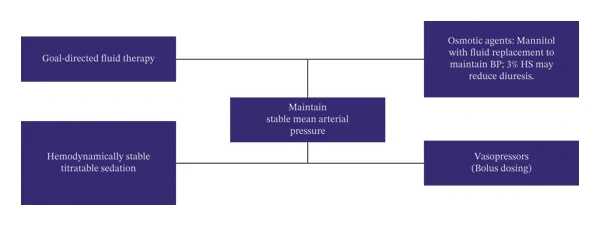
Management of intraoperative hypotension in awake craniotomy. Cerebral hypoperfusion.

Awake craniotomy rarely requires inhalational anesthetics, with only low‐dose IV sedatives used alongside scalp nerve blocks for analgesia. This approach minimizes the hypotension commonly associated with general anesthesia, reduces the need for vasopressors, and promotes more stable hemodynamic and physiological conditions overall [53]. Dexmedetomidine’s *α*2‐adrenergic agonism provides sedation without respiratory depression, making it ideal for hypotension prevention in awake craniotomy [53]. Remimazolam, an ultrashort‐acting benzodiazepine, offers stable hemodynamics with fewer hypotensive events and rapid, reversible recovery, making it a promising anesthetic option for awake craniotomy (Figure 3) [54]. Goal‐directed fluid therapy, utilizing stroke volume variation and other dynamic indices, can optimize preload while minimizing fluid overload in awake patients undergoing craniotomy [55]. Mannitol (0.5–1.0 g/kg) requires individualized dosing based on patient volume status and renal function, with concurrent fluid replacement to prevent hypotension [56]. While there is limited evidence comparing hypertonic saline (3% HS) with mannitol (20%) in awake craniotomy, RCTs in elective supratentorial tumor surgery suggest HS provides equal or superior brain relaxation with less diuresis. In clinical practice, HS may therefore be considered a potential alternative in awake procedures, although this remains an expert opinion pending validation specific to awake craniotomy [57, 58].

#### 4.3.2. Vasopressor Therapy

In the Glostrup Hospital series of awake craniotomies, intraoperative hypotension was reported in about 5% of cases. When it occurred, management involved the use of vasopressors, specifically ephedrine and phenylephrine, to restore blood pressure and maintain hemodynamic stability [59]. Case evidence further supports bolus therapy (Figure 3), with hypotension successfully corrected using repeated doses of ephedrine (4 mg) and phenylephrine (0.1 mg), underscoring the role of targeted vasopressor boluses rather than continuous infusion in maintaining hemodynamic stability [60].

#### 4.3.3. Case‐Based Management Approaches

In an observational study on dexmedetomidine sedation for awake craniotomy, hypotension was managed by reducing the infusion rate, with blood pressure returning to normal within minutes. In cases of hypotension due to sagittal sinus bleeding, treatment included intravenous fluids and mephentermine. Separate episodes of transient hypotension with bradycardia occurred independently of drug effects [27].

### 4.4. Evidence on Intraoperative Hypotension Management

Intraoperative hypotension during awake craniotomy represents a critical but under‐recognized determinant of neurological outcomes. Although it is usually iatrogenic, resulting from factors, such as anesthetic depth, sympathetic blockade, osmotic diuretics, or blood loss, it can also arise from procedure‐related causes, such as sagittal sinus bleeding or excessive cerebrospinal fluid drainage. Current evidence consistently links even brief MAP reductions (< 65 mmHg) to postoperative stroke and cognitive decline, yet no consensus exists regarding optimal thresholds or duration limits. Despite the availability of *α*_2_‐agonist‐based sedation and scalp blocks that minimize hypertensive surges, paradoxical hypotension remains common because of reduced sympathetic tone and impaired autoregulation in patients with gliomas or prior cerebrovascular disease.

The heterogeneity of anesthetic techniques, fluid strategies, and vasopressor algorithms across institutions prevents the development of standardized management pathways. Most studies remain observational, small, and inadequately powered to distinguish anesthetic‐induced from procedure‐induced hypotension. Future studies should aim to elucidate these mechanisms and establish evidence‐based intervention targets by incorporating multimodal monitoring techniques, including near‐infrared spectroscopy (NIRS), diffuse correlation spectroscopy (DCS), and cerebral microdialysis (CMD), to link perfusion metrics with neurological outcomes. Randomized comparisons of sedative regimens (dexmedetomidine, propofol, and remimazolam) are needed to identify the agent that best preserves cerebral perfusion while maintaining patient cooperation.

Ultimately, developing closed‐loop or artificial intelligence (AI)–driven systems capable of dynamically adjusting fluid and vasopressor infusions in response to real‐time cerebral perfusion data could provide more consistent control of CBF and reduce ischemic risk. Establishing validated CPP and MAP thresholds, together with cost‐effectiveness analyses of enhanced hemodynamic protocols, should form key priorities for future multicenter studies aimed at standardizing intraoperative hypotension management during awake craniotomy.

## 5. Cerebral Hypoperfusion and Ischemic Risk

### 5.1. Triggers and Physiological Relevance

**FIGURE 4:**
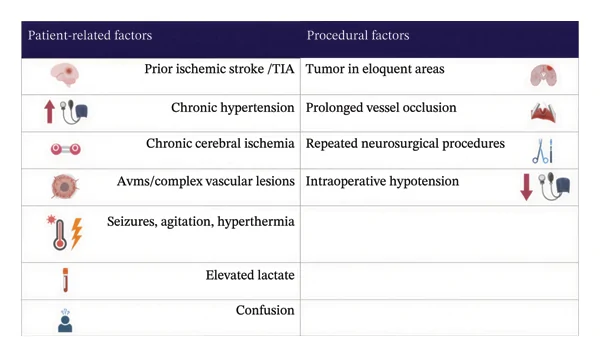
Key patient‐related and surgical/procedural factors contributing to cerebral hypoperfusion during awake craniotomy.

Key risk factors for cerebral hypoperfusion could be divided into two major groups, as shown in Figure 4:

Among patient‐related factors, prior ischemic stroke and a history of transient ischemic attack represent well‐established risks [61]. Chronic hypertension, which leads to impaired vascular compliance, further contributes to ischemic risk [62]. In addition, structural abnormalities, such as arteriovenous malformations (AVMs) or other complex vascular lesions, can significantly disrupt regional blood flow [63]. Chronic cerebral ischemia is another trigger. In such cases, the accumulation of reactive oxygen species (ROS), glutamate, and other vasoactive molecules has been shown to cause progressive neuronal damage [64]. Conditions of high metabolic demand (e.g., seizures, agitation, and hyperthermia) can increase susceptibility to hypoperfusion. Biochemical markers provide important understandings: For example, elevated lactate in blood serum exceeding 2.2 mmol/L has been associated with an increased risk of cerebral hypoperfusion (14.7% vs. 7.3%, *p* = 0.015) and developing new neurological deficit (odds ratio [OR] 2.11; 95% confidence interval [CI], 1.09–4.11; *p* = 0.028) [63]. Finally, clinical manifestations, such as confusion, may serve as critical warning signs. In fact, confusion during the awake phase of surgery has been strongly linked to ischemic injury: One study found that 29% of patients with cerebral infarction developed intraoperative confusion, compared to 0% of the patients without a diagnosed infarct (*p* = 0.023) [61].

Surgical and procedural factors include tumor infiltration in eloquent areas. This condition of the brain often requires complex, prolonged resections with meticulous cortical and subcortical mapping. The increased surgical complexity raises the risk of inadvertent hypoperfusion or direct vascular injury [65]. Another important contributor is prolonged temporary vessel occlusion. Temporary clipping or prolonged vessel occlusion during complex tumor resections during awake craniotomy can result in significant reductions in regional CBF [66]. Intraoperative hemodynamic instability must be considered. Intraoperative hypotension has been strongly associated with decreased cerebral perfusion and poorer neurological outcomes [67]. Finally, the risk of cerebral ischemia is increased in patients undergoing repeated neurosurgical procedures. Scar tissue formation combined with compromised collateral circulation has the potential to exacerbate susceptibility to hypoperfusion after repeated neurosurgical procedures [61].

### 5.2. Clinical Implications

#### 5.2.1. Cerebral Perfusion and Autoregulation

CBF is regulated by autoregulation, which maintains stable perfusion despite fluctuations in systemic blood pressure. Cerebral perfusion pressure (CPP) is the difference between the MAP and the ICP, measured in millimeters of mercury (mm Hg). An optimal CPP is 60–80 mmHg, with 60–70 mmHg recommended during awake neurosurgical procedures to minimize the risk of ischemia [61]. Ischemic events can disrupt the BBB, contributing to neurological deficits and long‐term functional impairments [64]. Moreover, conditions, such as chronic hypertension, prior ischemic stroke, and local tumor spreading, cause the microvascular changes that disrupt autoregulation of the brain [62].

The most reported perioperative complications can be categorized into acute and delayed events. Acute perioperative complications include seizures, reported in 2.6%–24% of cases, which are strongly associated with direct electrocortical stimulation during awake craniotomy [19, 28, 68]. Additional risk factors for perioperative seizures include a prior history of epilepsy (OR, 2.18; 95% CI, 1.07–4.46; *p* = 0.03), the presence of a brain tumor (OR, 2.41; 95% CI, 1.16–4.19; *p* = 0.02), and undergoing temporal craniotomy (OR, 5.18; 95% CI, 2.03–13.25; *p* = 0.001) [69]. Nausea and vomiting, affecting approximately 7% of patients, represent another common complication [70] and are often associated with the combined use of propofol and remifentanil (PR) anesthesia [30]. New neurologic deficits occur in 7.3%–16% of cases [63, 71] and are frequently related to the location of the brain tumor [72, 73]. Agitation, observed in approximately 6% of patients, is another perioperative complication associated with awake neurosurgical procedures [44]. Delayed complications include cognitive dysfunction. In the early postoperative period, psychomotor function was most affected by awake brain surgery [73]. In 50% of cases, mild memory and executive function impairment were observed [74]. Severe neurologic deficit was reported in 33% of patients, with improvement to 9% at the 6‐month follow‐up period, and 3% at 12 months [75].

A follow‐up study showed that the majority of postoperative neurological deficits improved over time, with resolution in most patients by the 12‐month follow‐up [76]. The lowest recovery in motor function over a 1‐year period was observed in acute ischemic stroke patients, from 58% to 37% [61].

### 5.3. Evidence‐Based Management/Neuroprotection as Shown in Figure 5

#### 5.3.1. Temperature Modulation

While general hypothermia showed no significant difference over normothermia in improving neurologic outcome (RR 0.80, 95% CI 0.61 to 1.04, *p* = 0.09) or reducing cerebral ischemia (RR 0.93, 95% CI 0.82 to 1.05, *p* = 0.24) during awake craniotomy [77], local cortical cooling (CC) during brain mapping showed 0% intraoperative seizures compared to standard electrical cortical stimulation (ECS) alone, which reported 3.6% [78].

**FIGURE 5:**
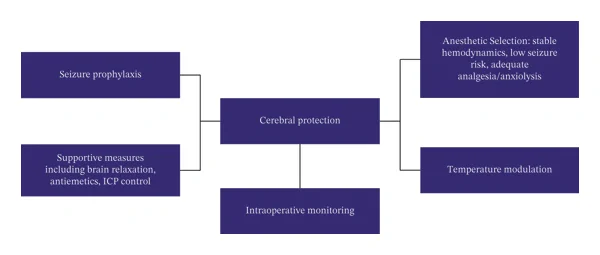
Integrated strategies for neuroprotection during awake craniotomy.

#### 5.3.2. Seizure Prophylaxis

The use of prophylactic anticonvulsants (phenytoin, fosphenytoin, and levetiracetam) significantly reduces risk of intraoperative seizures (phenytoin/fosphenytoin: OR, 0.12; 95% CI, 0.04–0.35; *p* < 0.001 and levetiracetam: OR, 0.40; 95% CI, 0.17–0.94; *p* = 0.04), while phenytoin/fosphenytoin showed to be more protective (OR, 0.31; 95% CI, 0.10–0.99; *p* = 0.048) [69].

#### 5.3.3. Pharmacological Strategies

Maintaining adequate cerebral perfusion without significantly disrupting metabolic and electrical activity is crucial for cerebral protection. Xenon anesthesia provides hemodynamic stability and does not affect baroreflex gain, and it decreases cerebral metabolism better than propofol [79]. The use of remimazolam anesthesia has also been shown to be effective in maintaining cerebral perfusion and metabolism and to facilitate faster arousal, making it a good option for awake craniotomy [54]. PR combinations showed reduced seizure incidence and fewer agitation events, but compared to the combination of propofol and dexmedetomidine (PD), PR had more frequent occurrence of nausea, vomiting, and oxygen desaturation (*p* < 0.05) [30]. Anesthetic management with continuous infusion of dexmedetomidine was documented to be superior to propofol, requiring fewer vasoactive agents and better compliance with intraoperative tests. It also reduces the risk of delirium and postoperative cognitive dysfunction [14]. Fewer intraoperative seizures were noted in the dexmedetomidine group (RR 4.26, 95% CI 1.49–12.16) [8], suggesting the neuroprotective properties of this drug. Other pharmacologic supportive measures for neuroprotection might include the administration of 4.95 mg IV dexamethasone during the induction phase, which has been noted to reduce the incidence of vomiting during awake surgery [70]. The infusion of hypertonic saline (3% NaCl) in moderate doses (3 mL/kg) provides better brain relaxation and ICP control compared to mannitol, with a good safety profile [80].

#### 5.3.4. Integrated Approach

There are several techniques that could help with effective intraoperative period and cerebral oxygenation monitoring. Preoperative diffusion tractography (DT) was shown to localize the tract with a sensitivity of ∼92%. Although the function of the tract could not be identified, this method has the potential to help reduce intra‐ and postoperative neurological complications and to predict recovery time [65]. ICG‐video angiography (ICG‐VG) is useful to detect cerebral perfusion changes and reduce operative time during mapping of eloquent areas, thereby enhancing the precision and reliability [81]. NIRS detected > 20% desaturation from baseline in only ∼15% of patients, suggesting limited sensitivity for cerebral perfusion monitoring when used as a single method [82]. Brain tissue oxygenation monitoring (brptiO2/PaO2 ratio) is more reliable than absolute brptiO2 readings for early hypoxia detection, especially in AVM resections [83]. Ultrafast Power Doppler Imaging (UPDI): A study demonstrates UPDI’s capacity for real‐time, high‐resolution monitoring of cerebral perfusion during events, such as temporary vessel clipping [84]. As a supplemental method for assessing depth of anesthesia, bispectral index (BIS) monitoring helps predict faster awakening and smoother transitions during asleep–awake procedures [82].

#### 5.3.5. Strategies for Optimizing Cerebral Perfusion

The key challenge that affects neurological outcomes during awake craniotomy and recovery remains cerebral hypoperfusion. While studies identify risk factors, such as hypertension, prior stroke, and intraoperative hypotension, there is no standardized protocol for maintaining stable cerebral perfusion. Sedation with *α*_2_‐agonist (dexmedetomidine) is suggested to be more effective in hemodynamic control, compared to the PR combination. The newer agent remimazolam shows potential for use but lacks strong evidence.

Future studies should compare sedative regimens (dexmedetomidine, propofol, and remimazolam) in controlled trials and establish safe blood pressure targets to improve patient outcomes using multimodal cerebral monitoring, such as NIRS, DT, or UPDI. These techniques can detect early perfusion and guide real‐time interventions. Future research should also explore automated or AI‐assisted systems to maintain cerebral perfusion more consistently during awake surgeries.

## 6. Future Directions and Recent Advances

### 6.1. Current Methods

MAC protocols maintain patient comfort while preserving cooperation for intraoperative testing. Evidence indicates that remifentanil reduces neurological deficits, propofol lowers seizure risk, and midazolam decreases agitation, while dexmedetomidine provides hemodynamic stability with minimal respiratory depression. These regimens, combined with scalp nerve blocks, local anesthetic infiltration, continuous physiological monitoring, and active patient participation, represent the standard [28].

Following establishment of MAC as the standard approach, intraoperative monitoring during awake craniotomy has relied primarily on MAP and BIS as global surrogate measures of cerebral perfusion and anesthetic depth, respectively. MAP remains the key actionable parameter for maintaining cerebral perfusion, with perioperative stroke guidelines emphasizing avoidance of hypotension and recommending MAP targets above approximately 70 mm Hg in patients at increased cerebrovascular risk [85]. In contrast, BIS is a processed frontal electroencephalographic (EEG) index used to guide hypnotic depth, with values between 40 and 60 commonly indicating adequate anesthesia and has been shown to reduce the risk of intraoperative awareness and facilitate recovery [86, 87]. Although BIS values may decrease during cerebral hypoperfusion due to EEG suppression, this relationship is indirect and insufficiently sensitive for ischemia detection, limiting its role to anesthetic titration rather than perfusion monitoring [88, 89]. Consequently, MAP and BIS should be interpreted as complementary but nonspecific global indicators, necessitating integration with more advanced monitoring modalities in neurosurgical practice.

To address these limitations, multichannel EEG systems, such as SedLine, employ four EEG channels across both hemispheres, enabling more reliable assessment of anesthetic depth and detection of burst suppression, particularly in older patients and those with altered EEG patterns. Updates to the Patient State Index (PSI) algorithm have reduced falsely elevated PSI values in elderly individuals, addressing a key limitation observed with earlier SedLine versions and single‐channel BIS monitoring [90]. However, SedLine indices remain subject to clinically relevant response delays and may underestimate the duration of EEG suppression compared with expert visual interpretation of raw EEG data [91, 92]. Consequently, SedLine offers improved reliability over BIS in complex neurosurgical cases but should be integrated within a multimodal monitoring strategy rather than used as a standalone decision‐making tool.

Emerging noninvasive tools, such as functional near‐infrared spectroscopy (fNIRS) and DCS, enable real‐time assessment of regional blood flow and neurovascular coupling. Transcranial Doppler (TCD) can be used to characterize autoregulatory status. These approaches remain largely investigational but may eventually integrate into multimodal platforms for more personalized neuroprotection strategies [93].

Current intraoperative hemodynamic management typically relies on static MAP targets and manual adjustments, which may not reflect rapid perfusion changes. The Pressure Reactivity Index (PRx), established in critical care, offers dynamic autoregulation assessment and estimation of individualized CPPopt, though its intraoperative use is limited. Additional metrics, such as rSO_2_ and PbtO_2_, could integrate perfusion and oxygen delivery. However, all remain investigational, as stable physiology is essential for functional mapping.

Neurotrophins (e.g., BDNF) and neuromodulator systems (dopamine and serotonin) have been implicated in long‐term reorganization. At present, these factors are best regarded as background considerations for preoperative stratification [94].

CMD can capture extracellular metabolites, offering early detection of ischemia or excitotoxic stress. While established in neurocritical care, intraoperative use remains rare and limited by sampling constraints [95].

Intraoperative connectomics, an emerging approach, shifts the focus from isolated cortical regions to preserving large‐scale brain networks that support complex functions, such as speech and movement during awake craniotomy. DT provides preoperative structural mapping of white‐matter pathways, which can be integrated into intraoperative neuronavigation. By contrast, intraoperative methods, such as DES, high‐density electrocorticography (ECoG), and task‐based testing, enable real‐time interrogation of functional connectivity. Experimental approaches using machine learning (ML) are being explored to synthesize these data streams. This strategy may support more personalized, function‐sparing resections near eloquent areas [96].

Preserving large‐scale brain networks, such as the default mode, salience, and frontoparietal control networks, better predicts cognitive outcomes than focusing solely on localized cortical regions. Intraoperative tools, such as high‐density ECoG and cortical stimulation, are now enabling real‐time assessment of these networks, moving surgery beyond tumor margins toward preserving distributed functional connectivity, especially in high‐risk cases (MK‐3) [97].

Expanding network preservation strategies, recent studies have examined the integration of neurocognitive testing with advanced electrophysiology to enhance intraoperative decision‐making. Task‐based monitoring of language, memory, and motor function remains standard, while high‐density ECoG provides additional spectral data, including gamma activity that may reflect network engagement. Preliminary evidence suggests that combining electrophysiological signals with patient performance may improve detection of subtle disruptions and guide tailored resections, though this approach remains investigational and requires further validation [97].

AI‐driven closed‐loop anesthesia systems integrate real‐time inputs, such as blood pressure, heart rate, and BIS, to automate the titration of anesthetic and vasoactive drugs. Evidence from general anesthesia suggests they may enhance hemodynamic stability and reduce variability. Recent prototypes incorporating multimodal feedback and ML highlight a conceptual pathway toward more adaptive control, though still experimental [98].

### 6.2. Cost‐Effectiveness Considerations

A recent time‐driven activity‐based costing analysis found that awake craniotomy incurred higher intraoperative costs than general anesthesia, largely due to personnel and supply utilization, with no significant difference in survival outcomes [21]. Together, these findings highlight that future adoption of advanced monitoring will depend on demonstrating not only clinical efficacy but also clear cost‐effectiveness across diverse healthcare settings.

### 6.3. Challenges

Patient factors, such as refusal, anxiety, or limited cooperation, restrict eligibility. Anesthetic challenges include airway obstruction, intraoperative seizures, nausea, hemodynamic instability, and risks of local anesthetic toxicity. Surgical difficulties, such as prolonged operative times, cerebral edema, significant blood loss, and logistical complications with intraoperative MRI, further increase the risk. At the system level, successful implementation requires highly skilled multidisciplinary teams, yet the scarcity of trained personnel remains a major limiting factor [21].

## 7. Role of AI and ML

Healthcare personnel are increasingly relying on AI models to identify hemodynamic instability and predict intraoperative hypotension in real time, which has been associated with decreased adverse outcomes [99]. Clinicians now have access to numerous AI tools, such as the Hypotension Prediction Index (HPI), which predicts impending hypotension before it occurs, enabling timely intervention rather than simply alerting after the MAP has already fallen below 65 mmHg [100, 101]. This is especially relevant in awake craniotomy as HPI can facilitate vasopressor or fluid management to shorten the duration and severity of hypotension as seen in a recent meta‐analysis of 43 studies and two randomized controlled trials [99].

ML models utilize calibrated algorithms to analyze PPG signals in conjunction with blood pressure cuff measurements, providing more accurate trending data compared to manual blood pressure readings [102]. ML models serve as a valuable supplement to clinicians, as shown in a retrospective study, where combining both arterial blood pressure (ABP) and electroencephalogram (EEG) waveforms improved the prediction of intraoperative hypotension over using single waveforms [103]. Current practice requires an expert to analyze EEG waves during an awake craniotomy to detect epileptic and seizure activity. ML‐automated EEG analysis methods show promise in expediting analysis to provide intervention during such episodes [104, 105]. This improvement is critical in awake craniotomy, where immediate recognition of hemodynamic instabilities during cortical mapping is essential. Advanced predictive monitoring models, such as those using combined ABP and EEG waveforms to forecast intraoperative hypotension, may enhance early detection of hemodynamic instability [103].

AI‐based systems serve as a valuable adjunct to anesthesiologists, particularly through closed‐loop control and clinical decision‐support platforms that assist with anesthetic and hemodynamic management [106]. These systems continuously integrate physiologic data to reduce variability in anesthetic depth and blood pressure compared with manual titration, thereby supporting more stable intraoperative conditions [106]. Although not specifically validated in awake craniotomy, such technologies are conceptually relevant to procedures requiring precise physiologic control, as excessive sedation or hemodynamic instability may compromise patient cooperation during cortical mapping [106]. In addition, AI‐enabled cognitive support systems can enhance situational awareness by generating timely alerts and suggesting tailored interventions, such as drug dose adjustments or fluid management strategies, while preserving clinician oversight [106].

Despite increasing interest in the use of AI in perioperative and neurosurgical practice, important concerns remain regarding clinician overreliance, appropriate calibration of trust, integration into existing workflows, and issues of accountability. These considerations highlight the need for formal training and clearly defined clinician competencies to ensure that AI‐based recommendations are interpreted appropriately and applied safely during awake procedures [107].

## 8. Limitations, Research, and Priorities

As a narrative review, this article was intentionally designed to provide a broad, integrative overview of intraoperative hemodynamic management and cerebral protection during awake craniotomy rather than a systematic synthesis of the literature. This approach was selected because the topic spans multiple domains, including anesthetic pharmacology, neuromonitoring, cerebrovascular physiology, functional mapping, and emerging technologies, such as AI, making a comprehensive narrative framework more appropriate for integrating diverse evidence. Consequently, this review does not employ formal systematic review methodology or quantitative meta‐analysis, and its conclusions should be interpreted within the context of the available evidence. Existing research on intraoperative hemodynamic management and cerebral protection during awake craniotomy is limited by several methodological and clinical challenges. Most studies are small‐scale, single‐center cohorts, retrospective analyses, or feasibility trials [108, 109]. The lack of multicenter, randomized controlled trials limits clinical interpretation and introduces bias. Future studies should therefore prioritize multicenter collaboration, standardized methodology, and adequately powered randomized designs to strengthen the evidence base.

Another major limitation is the inconsistent reporting of key clinical parameters across studies. Several investigations, including those by Saito et al. and Hansen et al., identified potential prognostic thresholds but did not consistently report follow‐up duration, tumor histology, or extent of resection, which limits interpretation and comparison across studies [108–110]. Future research would benefit from the adoption of standardized reporting practices that include tumor type and location, histologic grade, extent of resection, anesthetic approach, and predefined hemodynamic goals. Consistent inclusion of these variables would improve comparability and support more robust meta‐analytic synthesis. Interpretation of the existing literature is further complicated by substantial clinical heterogeneity. Blood pressure targets, fluid management strategies, and vasopressor use vary widely across institutions [111]. Definitions of hypotension and hypertension also varied across studies, with some using MAP‐based thresholds and others using systolic pressure‐based criteria, such as SAP or SBP. This heterogeneity limits direct comparison of incidence rates and outcomes across studies and prevents firm conclusions regarding a single optimal intervention threshold. Further study is needed to evaluate standardized ranges for MAP and CPP, such as maintaining MAP within approximately 75–95 mmHg and CPP between 60 and 70 mmHg. Comparative investigations should assess whether individualized targets based on tumor characteristics, surgical location, or patient comorbidities are associated with improved neurological outcomes.

Anesthetic variability further limits reproducibility. The current literature reflects the widespread use of SAS techniques, MAC, and various sedative regimens [68, 112]. Future trials should directly compare sedation protocols based on dexmedetomidine, propofol, and remimazolam with respect to hemodynamic stability, mapping success, cognitive recovery, and conversion to general anesthesia. Future research should also clarify the bidirectional relationship between functional mapping and hemodynamic instability, including whether real‐time mapping phases are associated with predictable blood pressure fluctuations that may influence mapping accuracy or postoperative neurological outcomes [2, 4, 18]. Likewise, alternative approaches, such as ultrasound‐guided scalp blocks, should be prospectively investigated in controlled clinical settings [45].

Population bias also restricts generalizability. Many studies have enrolled younger, healthier cohorts, with median ages under 40 years old [22]. Other population groups, such as elderly patients, individuals with chronic hypertension, obesity, elevated ICP, or previous craniotomies, remain underrepresented [113]. Future studies should incorporate these patient populations to better understand the impact of various comorbidities on hemodynamic stability and postoperative recovery.

Allocation bias represents another obstacle. Many comparative studies assign interventions based on surgeon or institutional preference rather than randomization [112]. Future research should utilize cluster‐randomized or stepped‐wedge study designs to reduce bias and assess the reproducibility of protocols across different resource environments. Variations between high‐ and low‐resource centers highlight the importance of developing tiered protocols that maintain patient safety while accounting for differences in available infrastructure [71].

Lastly, most existing literature focuses narrowly on intraoperative feasibility rather than long‐term, patient‐centered outcomes. Long‐term neurological function, seizure control, cognitive recovery, and quality of life are seldom assessed [112]. Future research should include a standardized core outcome set that captures both perioperative measures, such as hemodynamic stability, seizure incidence, and conversion rate, as well as long‐term outcomes, including neurocognitive performance, quality of life, and return to function. Establishing a multicenter registry would offer real‐time benchmarking of MAP/CPP targets, sedation protocols, and postoperative outcomes.

In summary, progress in this field depends on moving beyond descriptive, single‐center studies toward coordinated, multicenter research conducted under standardized protocols. Future studies should define evidence‐based hemodynamic thresholds, compare sedation regimens in controlled trials, validate multimodal cerebral monitoring methods against imaging and clinical endpoints, include high‐risk and older populations, and link intraoperative metrics to long‐term neurological outcomes. These priorities will help bridge existing knowledge gaps and support the development of reproducible, evidence‐based guidelines for intraoperative hemodynamic management and cerebral protection during awake craniotomy.

## Funding

No funding was received for this manuscript.

## Conflicts of Interest

The authors declare no conflicts of interest.

## Data Availability

Data sharing is not applicable to this article as no datasets were generated or analyzed during the current study.
